# Anserine is expressed in human cardiac and skeletal muscles

**DOI:** 10.14814/phy2.15833

**Published:** 2023-09-28

**Authors:** Lívia de Souza Gonçalves, Wagner Ribeiro Pereira, Rafael Pires da Silva, Guilherme Carvalho Yamaguchi, Victor Henrique Carvalho, Bianca Scigliano Vargas, Leonardo Jensen, Marisa Helena Gennari de Medeiros, Hamilton Roschel, Guilherme Giannini Artioli

**Affiliations:** ^1^ Applied Physiology & Nutrition Research Group—Center of Lifestyle, Faculdade de Medicina Universidade de São Paulo São Paulo Brazil; ^2^ Division of Pediatrics Department of Pediatrics University of California San Francisco California USA; ^3^ Departamento de Bioquímica, Instituto de Química Universidade de São Paulo São Paulo Brasil; ^4^ Laboratorio de Hipertensao do Instituto do Coraçao do Hospital das Clínicas da Faculdade de Medicina da Universidade São Paulo São Paulo Brazil; ^5^ Department of Life Sciences Manchester Metropolitan University Manchester UK

**Keywords:** anserine, carnosine‐N‐methyltransferase, heart, human, muscle

## Abstract

We evaluated whether anserine, a methylated analog of the dipeptide carnosine, is present in the cardiac and skeletal muscles of humans and whether the *CARNMT1* gene, which encodes the anserine synthesizing enzyme carnosine‐N‐methyltransferase, is expressed in human skeletal muscle. We found that anserine is present at low concentrations (low micromolar range) in both cardiac and skeletal muscles, and that anserine content in skeletal muscle is ~15 times higher than in cardiac muscle (cardiac muscle: 10.1 ± 13.4 μmol·kg^−1^ of dry muscle, *n* = 12; skeletal muscle: 158.1 ± 68.5 μmol·kg^−1^ of dry muscle, *n* = 11, *p* < 0.0001). Anserine content in the heart was highly variable between individuals, ranging from 1.4 to 45.4 μmol·kg^−1^ of dry muscle, but anserine content was not associated with sex, age, or body mass. We also showed that *CARNMT1* gene is poorly expressed in skeletal muscle (*n* = 10). This is the first study to demonstrate that anserine is present in the ventricle of the human heart. The presence of anserine in human heart and the confirmation of its expression in human skeletal muscle open new avenues of investigation on the specific and differential physiological functions of histidine dipeptides in striated muscles.

## 1 INTRODUCTION

The histidine‐containing dipeptide (HCD) anserine (β‐alanyl‐N‐π‐methyl‐L‐histidine) is one of the methylated analogs of carnosine (β‐alanyl‐L‐histidine). Carnosine, in turn, is found in several mammal species (Dolan et al., 2018; Flancbaum et al., 1990) and is expressed in striated muscles (Everaert et al., 2013,Saunders et al., 2017), kidneys (Peters et al., 2015), and some areas of the brain (Flancbaum et al., 1990). Carnosine is formed from its constituent amino acids L‐histidine and β‐alanine in a reaction catalyzed by the enzyme carnosine synthase (*CARNS1* gene) (Drozak et al., 2010). In humans, carnosine concentrations can vary depending on tissue, dietary ingestion of carnosine precursors, sex, and age (Crush, 1970; Drozak et al., 2015; Rezende et al., 2020). Increased intake of the amino acid β‐alanine via meat ingestion or supplementation has been shown to increase the carnosine content in the skeletal muscle by ~50%–100% (Rezende et al., 2020; Saunders et al., 2017). Skeletal muscle is the tissue where carnosine is most abundant, representing 99% of the total bodily pool of carnosine (Boldyrev et al., 2013). The biological importance of carnosine resides on its properties and purported functions, which include H^+^ buffering (Harris et al., 2006; Smith, 1938), regulation of Ca^2+^ transients and sensitivity (Dutka et al., 2012), protection against glycation and carbonylation, and detoxification of reactive aldehydes (Carvalho et al., 2018).

Anserine on the other hand is far less studied than carnosine, and little is known about this dipeptide. Anserine synthesis is catalyzed by the carnosine‐N‐methyltransferase enzyme (*CARNMT1* gene), and it occurs by transferring a methyl group from S‐adenosylmethionine onto carnosine to form anserine (Drozak et al., 2015; McManus, 1962). Anserine synthesis, therefore, depends on the prior synthesis of carnosine. This was confirmed by a study with *CARNS1* knockout rats (i.e., unable to synthesize carnosine), which showed that these animals do not express any anserine in their tissues (Goncalves et al., 2021). Some studies suggested that anserine has similar properties to carnosine, acting as a proton (H^+^) buffer, antioxidant, radical scavenger, and Ca^2+^ handling regulator (Abe et al., 1985; Batrukova & Rubtsov, 1997; Kohen et al., 1988), but the physiological functions of anserine and its importance to homeostasis remain largely unknown.

While anserine is highly expressed in the skeletal and cardiac muscles of several species of mammals and avians (Christman, 1976; Dolan et al., 2018; Everaert et al., 2013), several studies reported that anserine is not present in human striated muscles (Boldyrev et al., 2013; Christman, 1976; Mannion et al., 1992). Likewise, the gene encoding the anserine‐forming enzyme, initially characterized in chickens as histamine‐N‐methyltransferase‐like protein (HNMT‐like) has been reported not to be part of mammalian genomes (Drozak et al., 2013). More recently, however, the mammalian ortholog of carnosine‐N‐methyltransferase was isolated from rat muscle, thoroughly characterized, and confirmed to catalyze anserine synthesis via methylation of carnosine (Drozak et al., 2015). The crystal structure of the human enzyme has been resolved a few years later (Cao et al., 2018). Drozak et al. (2015) showed that the *CARNMT1* gene is expressed in human skeletal muscle; however, samples from only two individuals were analyzed thereby warranting further investigation on the expression of *CARNMT1* in muscle tissue.

Although carnosine has been considered the only HCD present in human skeletal muscle (Peters et al., 2015), a more recent study has challenged the notion that anserine is not expressed in human skeletal muscle (Hoetker et al., 2018). In this investigation, anserine was found at very low concentrations (~0.1 nmol·g^−1^ of tissue), which is ~100 times less than the typical carnosine concentrations in human skeletal muscle. Furthermore, they showed that, similar to carnosine, muscle anserine levels increase in response to β‐alanine supplementation (~20% increase), as previously shown in mice (Everaert et al., 2013). To the best of our knowledge, this was the only study to show the presence of anserine in human skeletal muscle, and so further confirmation is necessary. Moreover, it is also unknown whether anserine is present in human cardiac muscle. While carnosine has been recently implicated in important regulatory and protective functions in cardiac muscles (Creighton et al., 2022; Goncalves et al., 2021; Zhao et al., 2020), whether anserine is present or displays any physiological function in the human heart remains unknown.

Considering the contrasting data on anserine in human skeletal and the lack of information on anserine in human cardiac muscle, we aimed to gather further knowledge on whether anserine is present in human striated muscles and to explore the expression of the *CARNMT1* gene in human skeletal muscle.

## 2 MATERIALS AND METHODS

### 2.1 Human skeletal muscle

*Vastus lateralis* muscle samples from healthy, physically active omnivorous males who took part in two studies previously conducted in our laboratory (Goncalves et al., 2020; Yamaguchi et al., 2021) were used. Samples from 11 individuals (age = 28 ± 5 years, body mass = 80.7 ± 6.5 kg, BMI = 24.7 ± 2.1 kg/m^2^) were used for HCDs determination whereas samples from 10 unrelated individuals (age = 28 ± 6 years, body mass = 71.3 ± 14.2 kg, BMI = 23.4 ± 2.6 kg/m^2^) were used for mRNA extraction and gene expression analysis. The samples used for HCDs analysis had been previously lyophilized for 16 h in a bench top freezer drier (Edwards Vacuum) and deproteinized for metabolite quantification, for which reason we could not use them for mRNA analysis. All samples were collected from the middle portion of the vastus lateralis using the percutaneous needle biopsy technique with suction (Goncalves et al., 2020, Yamaguchi et al., 2021) and were snap frozen immediately after collection and preserved at −80°C for later analysis. All experiments were approved by the Research Ethics Committee of the University of Sao Paulo and complied with the standards established by the Declaration of Helsinki (#1942548 and #1185971). All participants provided written informed consent before taking part in the studies.

### 2.2 Postmortem human heart

Samples from segments of the ventricular wall from 12 individuals who deceased from different causes were donated and used in this study (Table 1). The samples were stored in a biorepository of the University of Sao Paulo in the vapor phase of liquid nitrogen and a small aliquot was transferred to our laboratory, where they were lyophilized for 16 h in a bench top freezer drier (Edwards Vacuum) and processed for anserine analysis as described below. Since the collection of human heart samples was not designed to preserve mRNA integrity, these samples were not submitted to mRNA analysis. The experiment was approved by the Research Ethics Committee of the University of Sao Paulo (#4802079).

**TABLE 1:** Individual characteristics of the donors of human heart samples used in this study.

ID	Age (years)	Sex	Body mass (kg)	BMI (mg∙kg^−2^)	Cause of death
1	11	F	38	NA	Acute respiratory failure
2	55	M	49	20.5	Malignant neoplasm
3	38	M	76	23.5	Cardiac failure and bacterial pneumonia
4	62	F	54	21.4	Acute respiratory failure
5	68	M	80	28.3	Cardiac failure and mesenteric ischemia
6	66	M	88	29.4	Pneumonia and septic shock
7	48	M	74	25.9	Malignant neoplasm
8	77	F	60	22.6	Pneumonia and septic shock
9	13	F	NA	NA	Acute respiratory failure
10	60	M	59	20.2	Cerebral vascular accident
11	74	F	56	23.9	Pneumonia and septic shock
12	66	M	66	22.8	Cardiac failure and bacterial pneumonia

Abbreviation: NA, not available.

### 2.3 Quantification of anserine and carnosine by liquid chromatography‐electrospray ionization‐tandem mass spectrometry (HPLC‐ESI^+^‐MS/MS)

Approximately ~5 mg of lyophilized skeletal and cardiac muscle tissues was powdered and deproteinized with 0.5 M HClO_4_, vortexed for 15 min, and centrifuged at 5000*g* at 4°C for 3 min (Dunnett & Harris, 1997). Samples were neutralized with 2.1 M KHCO_3_, centrifuged at 5000*g* at 4°C for 3 min, and the supernatant was stored at −80°C for further analysis. The samples were quantified in duplicate by online HPLC‐ESI^+^‐MS/MS using CAR*d*_*4*_ as internal standard, as described before (Goncalves et al., 2020). Briefly, the analyses were carried out in positive mode on a triple quadrupole mass spectrometer API 6500 (Sciex) using selected reaction monitoring (SRM). For sample injection and cleanup, was used an Agilent HPLC system (Agilent Technologies) equipped with an autosampler (1200 High performance), a column oven set at 45°C (1200 G1216B), an automated high pressure flow switching valve, a 1200 Binary Pump SL, and a Shimadzu10‐AVp Isocratic Pump (Shimadzu) on two Kinetex C18 columns (Phenomenex). At the mass spectrometer, nebulizer and auxiliary gas were set to 40 psi and the Dwell Time used was 100 ms. The signal to noise ratio (S/N) ≥7 was used as the quantification criteria. The transitions used for anserine were: quantification transition (241 → 170 m/z), confirmation transition (241 → 109 m/z). The transitions used for carnosine were: quantification transition (227 → 110 m/z), confirmation transition (227 → 156 m/z). For CARd_4_ C the transitions were 231 → 110 m/z (quantification) and 231 → 156 m/z (confirmation).

### 2.4 Real‐time PCR

Real‐time PCR was used to determine the expression of the *CARNMT1* gene in human *vastus lateralis* having the *EEF1A1* gene as a reference. Total RNA was isolated using Trizol® (Invitrogen) and chloroform and precipitated in isopropanol. RNA concentration and purity were measured in a micro‐spectrophotometer (NanoDrop ND2000, Thermo Scientific), with integrity being confirmed in denaturing agarose gel. cDNA was synthesized with oligo DT and M‐MLV reverse transcriptase (Promega). PCR was carried out with 20 ng cDNA, 22 μL SYBR™ Green (Applied Biosystems), and 300 nM of each primer in a final volume of 44 μL. The cycling conditions were 50°C for 2 min, 95°C for 10 min, 40 cycles of 95°C for 15 s and 60°C for 60 s, and a final 65–95°C melting ramp with 1°C increments. Signal intensity was monitored using the Rotor Gene‐Q HRM system (Qiagen). Primers sequences were as follows: *CARNMT1*: 5′‐TTCCGCTACTACGGCACC‐3′ and 5′‐TCCGGATCTTGTCCAAGTGA‐3; *EEF1A1*: 5′‐CTGGCAAGGTCACCAAGTCT‐3′ and 5′‐CCGTTCTTCCACCACTGAT‐3′.

### 2.5 Statistical analysis

Data were confirmed to be normally distributed using the Shapiro–Wilk test. Welch two‐samples *t*‐tests were used to compare differences in cardiac anserine content between males and females, and between skeletal and cardiac muscles. Differences in carnosine versus anserine in skeletal muscle were compared using paired‐sample *t*‐test. Pearson product–moment correlation analysis was used to explore whether heart anserine content is associated with age or body mass. All analyses were conducted in the R‐Studio software v.4.0.3. Data are presented as mean ± SD and the alpha level was previously set at 5%.

## 3 RESULTS

### 3.1 Anserine content in cardiac muscle

**FIGURE 1:**
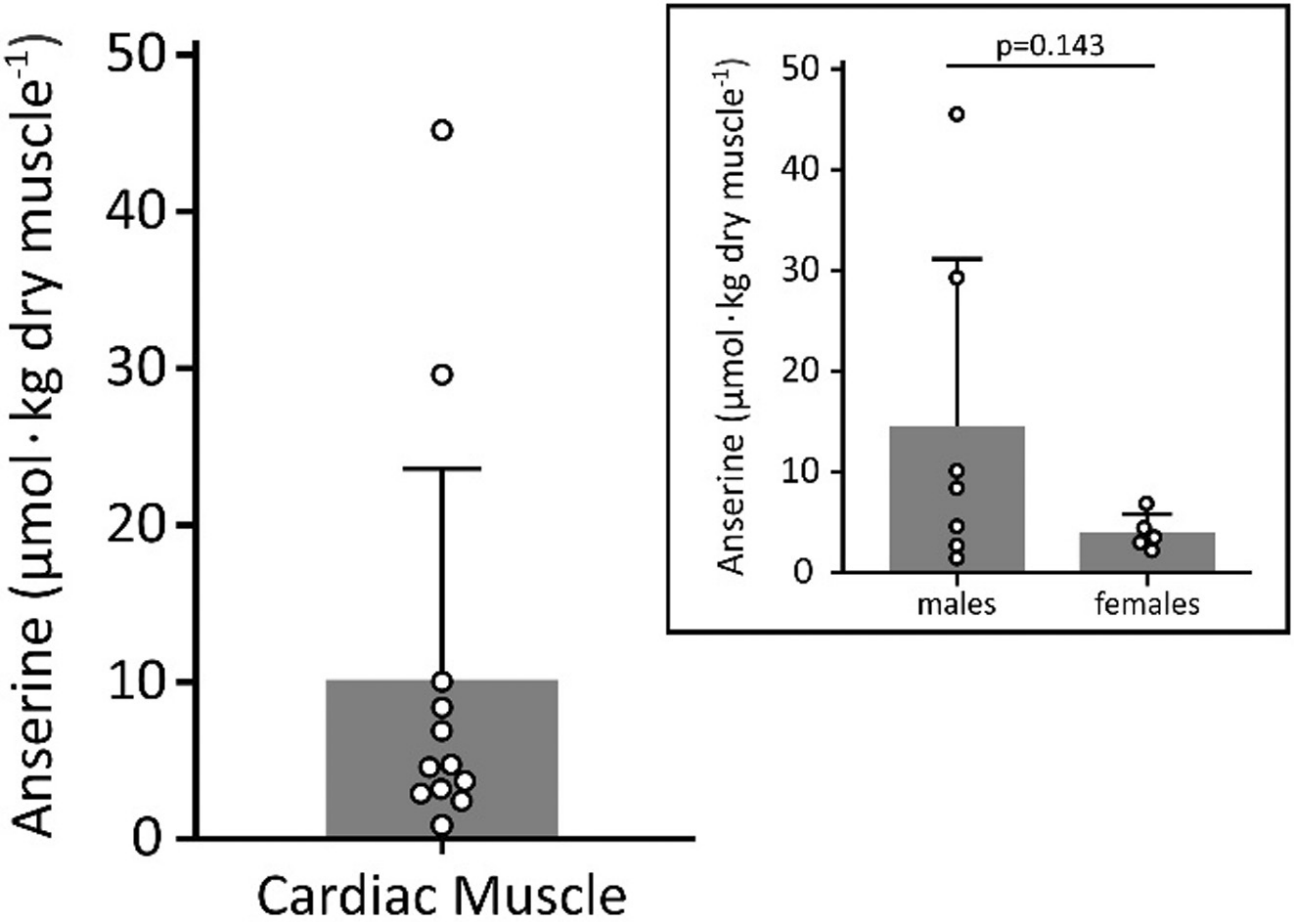
Anserine content in ventricle postmortem samples obtained from 12 donors. The same dataset is broken down into male and female donors (offset chart).

We observed very low concentrations of anserine in human heart ventricle, sitting in the low micromolar range (Figure 1). A considerably large interindividual variation was observed, with values ranging from ~1.5 to 45 μmol·kg^−1^ dry tissue. Larger variation was observed in the male samples compared to females, but no significant differences were observed between sexes (Figure 1). This variation was not associated with age (*r* = −0.16, *p* = 0.62) or body mass (*r* = 0.42, *p* = 0.19).

### 3.2 HCDs content in skeletal muscle

**FIGURE 2:**
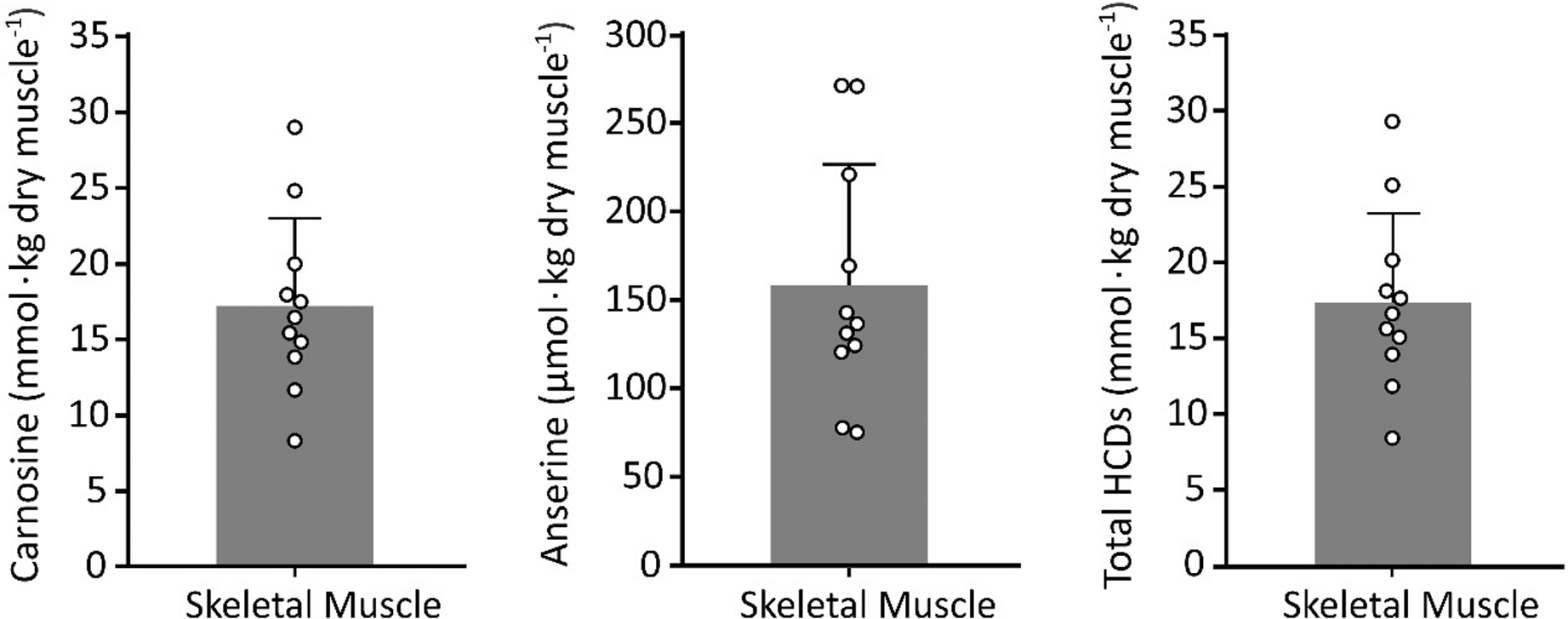
Carnosine, anserine, and total HCDs (carnosine + anserine) in human skeletal muscle.

Anserine was also observed in the m. *vastus lateralis* samples (all male participants), however at ~15 times higher levels compared to the heart (*p* < 0.0001). Yet, the skeletal muscle anserine content sits in the micromolar range while carnosine content sits in the millimolar range (*p* < 0.0001); anserine therefore, contributes only minimally to the total HCDs content in skeletal muscle (Figure 2).

### 3.3 CARNMT1 expression

**FIGURE 3:**
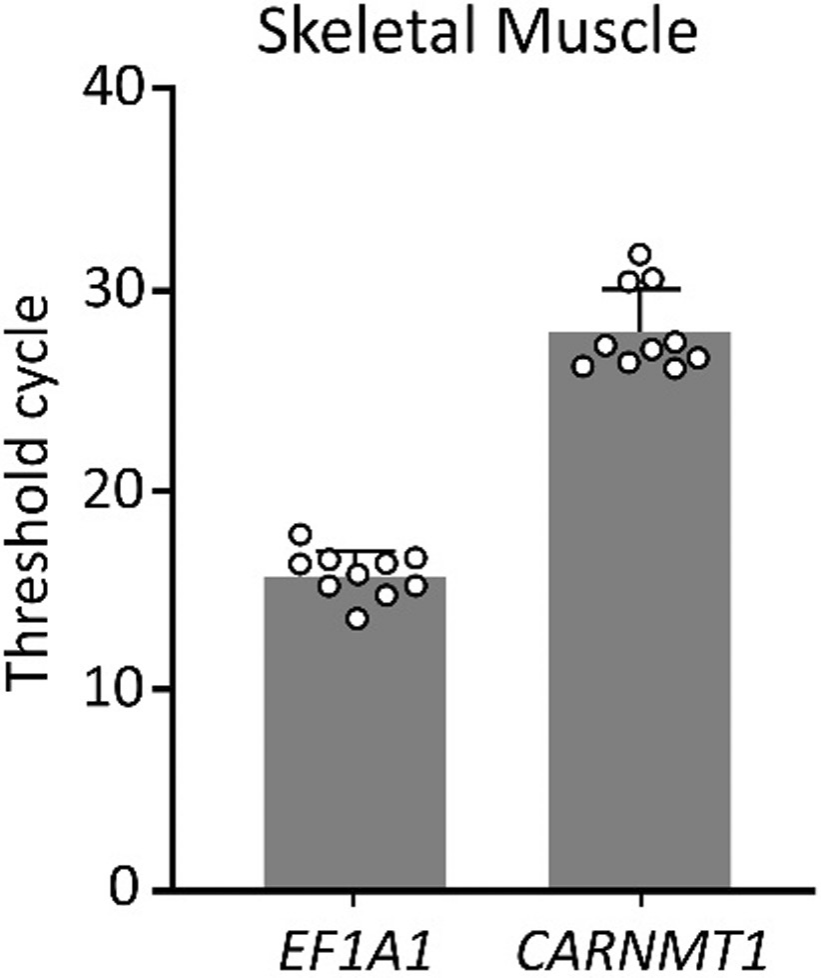
Expression of the genes *EF1A1* (reference gene) and the *CARNMT1*, expressed as number of cycles required to reach fluorescent signal threshold.

Gene expression analysis of the *CARNMT1* gene confirmed that it is expressed in human skeletal muscle; however, the expression levels are comparatively low in relation to the *EF1A1* reference gene (all male participants, Figure 3).

## 4 DISCUSSION

It is widely known that carnosine is abundantly expressed in human skeletal muscle (Rezende et al., 2020). Carnosine also seems to be present in the cardiac muscle of humans (Flancbaum et al., 1990) and other mammals (Blancquaert et al., 2016), although much less information is available on this matter. Until recently, it was believed that carnosine was the sole HCD in human striated muscles. However, in 2018, Hoetker et al. used a highly sensitive analytical method and reported that anserine is expressed in human skeletal muscle. Here, using a similarly sensitive analytical technique, we confirmed that anserine is present in human skeletal muscle and further expanded this notion to human cardiac muscle. This indicates that it is time to change the old view that carnosine is the only HCDs in human striated muscle. We also reported that anserine content is ~100 times lower than carnosine in the skeletal muscle (0.16 ± 0.07 and 17.27 ± 5.79 mmol·kg^−1^ dm, respectively) and that the *CARNMT1* gene is expressed in the human skeletal muscle.

Since anserine levels in skeletal muscle are too close or below the lowest detection limits of most liquid chromatography methods, it is likely that previous studies using these methods were unable to detect anserine in human muscle. The discrepancy in the literature, therefore, seems to have been caused by the lower sensitivity of the methods used. This indicates that future studies focusing on anserine metabolism in humans should use more sensitive techniques such as mass spectrometry or other methods optimized for anserine quantification.

One important question that arises from the data here reported is why anserine content is so much lower than carnosine content in skeletal muscle. We propose that this results from a combination of three factors, as follows: (1) the low expression of the *CARNMT1* gene, as shown in this study; (2) the low catalytic efficiency of the carnosine‐N‐methyltransferase enzyme, as demonstrated by Drozak et al. (2010); (3) the fact that anserine synthesis relies on the synthesis of carnosine in the first place. In relation to the relevance of *CARNMT1* gene expression levels, both *CARNMT1* (Drozak et al., 2015) and anserine (Peters et al., 2015) were previously reported to be expressed in human kidney, with *CARNMT1* mRNA levels being ~10 times higher in kidney than in muscle (Drozak et al., 2015) and the anserine concentration in the kidney also being ~10 times higher (1.1–7.4 mmol·kg^−1^ dry tissue, (Peters et al., 2015) compared to what is shown in skeletal muscle in this study and in Hoetker et al. (2018). In relation to anserine synthesis being dependent on the previous synthesis of carnosine, carnosine methylation seems to be the sole pathway for anserine formation. This is supported by studies with humans (Hoetker et al., 2018), mice (Everaert et al., 2013), and cell culture (Bauer & Schulz, 1994) that showed increased anserine following β‐alanine supplementation, an intervention that knowingly increases carnosine content (Harris et al., 2006; Saunders et al., 2017). Since carnosine is a substrate of the carnosine‐N‐methyltransferase enzyme, increased carnosine is expected to result in increased anserine. To further support this notion, we have previously shown that *CARNS1* gene knockout (resulting in the complete absence of carnosine) also leads to the complete absence of anserine in rat skeletal and cardiac muscles (Goncalves et al., 2021). In a scenario of low expression and catalytic efficiency of the sole anserine‐forming enzyme where carnosine formation is the main substrate for anserine synthesis, it seems natural that anserine levels will necessarily fall far below carnosine levels. Finally, the fact that carnosine content in the cardiac muscle is lower than in the skeletal muscle (Boldyrev et al., 2013; Creighton et al., 2022) and that the same pattern was observed for anserine in this study seems reinforce the idea that carnosine levels have a strong influence on anserine levels.

This study is the first to show that anserine is expressed in human cardiac muscles. Importantly, this was done in a relatively large number of samples obtained from a diverse group of donors. Although a large interindividual variability in anserine content was observed, this was not significantly associated with sex, age, or body mass, despite previous studies indicating that sex and age can affect muscle carnosine content (Everaert et al., 2011; Lievens et al., 2021; Mannion et al., 1992). It is currently unclear whether these factors do not play a similar role in the cardiac muscle or whether our study is not adequately designed to detect such effects. Since the samples used in this study were sourced from an institutional biorepository, we have access to limited information about the donors and have no means to select their characteristics. Notably, most donors had underlying cardiac conditions, which could influence HCDs and anserine levels in the heart. Moreover, diet and meat ingestion are arguably the most influential factors affecting HCDs levels in skeletal muscle (Harris et al., 2012; Painelli et al., 2018; Yamaguchi et al., 2021), and possibly in other tissues. Since we have no information on the dietary habits of the donors, we cannot rule out the possibility of this large variation being caused, at least in part, by differences in meat ingestion.

Another important question that arises from our data is what function(s) anserine plays in the skeletal and cardiac muscles. Anserine has been far less studied than its analog carnosine, and little is known about its biological relevance. Nonetheless, a few studies have reported that anserine displays similar properties compared to carnosine, including similar pKa (7.1 and 6.83) (Abe, 2000; Harris et al., 2006), antioxidant activity, radical scavenger activity, and Ca^2+^ handling regulator (Abe et al., 1985; Batrukova & Rubtsov, 1997; Kohen et al., 1988). Despite the similarities in chemical properties, it seems counterintuitive to conceive that anserine and carnosine play similar biological functions. Proton buffering capacity is highly dependent on buffer concentration; therefore, it seems highly unlikely that anserine could be a relevant H^+^ buffer in human striated muscles being expressed in low micromolar levels. To date, there is no clear understanding of the regulatory functions of HCDs on cardiac muscle (Creighton et al., 2022). Studies using genetically modified animals indicated that HCDs are key regulators of excitation–contraction coupling in cardiomyocytes (Goncalves et al., 2021), and that they can confer protection to the heart tissue in conditions of increased stress (Zhao et al., 2020). The protection exerted by HCDs seems to be related to the H^+^ buffering action of HCDs when the heart is under acidosis secondary to ischemia, and due to their ability to quench reactive aldehydes via formation of adducts (Zhao et al., 2020). As discussed, anserine is unlikely to be a relevant H^+^ buffer; moreover, all reports of aldehyde detoxification indicate that carnosine can form adducts (Bispo et al., 2016; Carvalho et al., 2018; Hoetker et al., 2018), with currently no indication that anserine could also form adducts with aldehydes. Since the study with *CARNS1* gene knockout showed that HCDs modulate EC‐coupling via Ca^2+^ handling did not differentiate the effects of carnosine and anserine (Goncalves et al., 2021), this remains a possible role for anserine in cardiac muscle, although we emphasize that this is currently unknown. Our data showing that anserine is present in human muscles opens a new avenue of investigation and represents a first step forward in understanding the functions of anserine in striated muscles. There is a clear need for more studies aiming to identify the physiological importance and roles played by anserine in human striated muscles.

This study is not without limitations. One important limitation is that lack of diversity in the participants from whom skeletal muscle biopsies were obtained. We used samples from a homogenous group of young, healthy, omnivorous, physically active male individuals; although this controls for several potentially intervening variables, our results cannot be extrapolated to other populations, and so it remains to be confirmed whether anserine is present (and to what extent) in the skeletal muscle of individuals with different characteristics. Another limitation is the use of postmortem cardiac samples, whose collection was not designed to preserve mRNA integrity, thereby rendering them unsuitable for gene expression analysis. The lack of confirmation of *CARNMT1* gene expression in these samples is a limitation, although the presence of anserine itself in this tissue, along with the fact that carnosine‐N‐methyltransferase is the sole anserine‐forming enzyme, strongly indicates that both the *CARNMT1* gene and its respective protein are expressed in the cardiac muscle. For reasons beyond our control, we were also unable to confirm that the carnosine‐N‐methyltransferase protein is expressed in both tissues, which we acknowledge is a limitation of this study.

To conclude, we showed for the first time that anserine is present in the human cardiac muscle and confirmed previous reports that anserine is also expressed in human skeletal muscle. While the biological functions of anserine remain unknown, these findings point to new directions for future research as it is yet to be demonstrated whether carnosine, anserine and other HCDs have specific and differential functions in human skeletal and cardiac muscles.

## AUTHOR CONTRIBUTIONS

Livia de Souza Goncalves: Study conceptualization; project administration and execution; sample analysis; data analysis; data interpretation; writing—original draft; writing—review & editing. Wagner Ribeiro Pereira: Sample analysis; data interpretation; writing—review & editing. Rafael Pires da Silva: Participant recruitment; sample collection; sample analysis; writing—review & editing. Guilherme Carvalho Yamaguchi: Participant recruitment; sample collection; sample analysis; writing—review & editing. Victor Henrique Carvalho: Sample analysis; data interpretation; writing—review & editing. Bianca Scigliano Vargas: Sample analysis; data interpretation; writing—review & editing. Leonardo Jensen: Sample collection; data interpretation; writing—review & editing. Marisa Helena Gennari de Medeiros: Study conceptualization; management of mass spectrometry analyses; data interpretation; writing—review & editing. Hamilton Roschel: Study conceptualization; data interpretation; writing—original draft; writing—review & editing. Guilherme Giannini Artioli: Study conceptualization; overall project management and supervision; data analysis; data interpretation; writing—original draft; writing—original draft; writing—review & editing. All authors read and approved the final version of the manuscript.

## FUNDING INFORMATION

L.S.G: Coordenação de Aperfeiçoamento de Pessoal de Nível Superior (CAPES); W.R.P: São Paulo Research Foundation—FAPESP (#2020/08964‐2). R.P.S: São Paulo Research Foundation—FAPESP (#2017/23688‐9). G.C.Y: São Paulo Research Foundation—FAPESP (#2015/231762). B.S.V: São Paulo Research Foundation—FAPESP (#2019/24899‐9). H.R: São Paulo Research Foundation—FAPESP (#2019/25032‐9). M.H.G.M: CEPID Redoxoma: 2013/07937‐8. This study was financed in part by the Coordenação de Aperfeiçoamento de Pessoal de Nível Superior—Brasil (CAPES‐ Finance Code 001) and CNPq (Conselho Nacional de Desenvolvimento Científico e Tecnológico).

## CONFLICT OF INTEREST STATEMENT

All authors declare no competing interests in this study.

## Data Availability

Data will be made available on request.

## References

[phy215833-bib-0001] Abe, H. (2000). Role of histidine‐related compounds as intracellular proton buffering constituents in vertebrate muscle. Biochemistry (Mosc), 65, 757–765. 10951092

[phy215833-bib-0002] Abe, H. , Dobson, G. P. , Hoeger, U. , & Parkhouse, W. S. (1985). Role of histidine‐related compounds to intracellular buffering in fish skeletal muscle. The American Journal of Physiology, 249, R449–R454. 4051030 10.1152/ajpregu.1985.249.4.R449

[phy215833-bib-0003] Batrukova, M. A. , & Rubtsov, A. M. (1997). Histidine‐containing dipeptides as endogenous regulators of the activity of sarcoplasmic reticulum Ca‐release channels. Biochimica et Biophysica Acta, 1324, 142–150. 9059507 10.1016/s0005-2736(96)00216-7

[phy215833-bib-0004] Bauer, K. , & Schulz, M. (1994). Biosynthesis of carnosine and related peptides by skeletal muscle cells in primary culture. European Journal of Biochemistry, 219, 43–47. 8307008 10.1111/j.1432-1033.1994.tb19912.x

[phy215833-bib-0005] Bispo, V. S. , de Arruda Campos, I. P. , Di Mascio, P. , & Medeiros, M. H. (2016). Structural elucidation of a carnosine‐acrolein adduct and its quantification in human urine samples. Scientific Reports, 6, 19348. 26783107 10.1038/srep19348 PMC4726056

[phy215833-bib-0006] Blancquaert, L. , Baba, S. P. , Kwiatkowski, S. , Stautemas, J. , Stegen, S. , Barbaresi, S. , Chung, W. , Boakye, A. A. , Hoetker, J. D. , Bhatnagar, A. , Delanghe, J. , Vanheel, B. , Veiga‐Da‐Cunha, M. , Derave, W. , & Everaert, I. (2016). Carnosine and anserine homeostasis in skeletal muscle and heart is controlled by beta‐alanine transamination. The Journal of Physiology, 594, 4849–4863. 27062388 10.1113/JP272050 PMC5009790

[phy215833-bib-0007] Boldyrev, A. A. , Aldini, G. , & Derave, W. (2013). Physiology and pathophysiology of carnosine. Physiological Reviews, 93, 1803–1845. 24137022 10.1152/physrev.00039.2012

[phy215833-bib-0008] Cao, R. , Zhang, X. , Liu, X. , Li, Y. , & Li, H. (2018). Molecular basis for histidine N1 position‐specific methylation by CARNMT1. Cell Research, 28, 494–496. 29463897 10.1038/s41422-018-0003-0 PMC5938892

[phy215833-bib-0009] Carvalho, V. H. , Oliveira, A. H. S. , de Oliveira, L. F. , da Silva, R. P. , Di Mascio, P. , Gualano, B. , Artioli, G. G. , & Medeiros, M. H. G. (2018). Exercise and beta‐alanine supplementation on carnosine‐acrolein adduct in skeletal muscle. Redox Biology, 18, 222–228. 30053728 10.1016/j.redox.2018.07.009 PMC6077140

[phy215833-bib-0010] Christman, A. A. (1976). Factors affecting anserine and carnosine levels in skeletal muscles of various animals. International Journal of Biochemistry, 7, 519–527.

[phy215833-bib-0011] Creighton, J. V. , de Souza Goncalves, L. , Artioli, G. G. , Tan, D. , Elliott‐Sale, K. J. , Turner, M. D. , Doig, C. L. , & Sale, C. (2022). Physiological roles of carnosine in myocardial function and health. Advances in Nutrition, 13, 1914–1929. 35689661 10.1093/advances/nmac059 PMC9526863

[phy215833-bib-0012] Crush, K. G. (1970). Carnosine and related substances in animal tissues. Comparative Biochemistry and Physiology, 34, 3–30. 4988625 10.1016/0010-406x(70)90049-6

[phy215833-bib-0013] Dolan, E. , Saunders, B. , Dantas, W. S. , Murai, I. H. , Roschel, H. , Artioli, G. G. , Harris, R. , Bicudo, J. , Sale, C. , & Gualano, B. (2018). A comparative study of hummingbirds and chickens provides mechanistic insight on the histidine containing dipeptide role in skeletal muscle metabolism. Scientific Reports, 8, 14788. 30283073 10.1038/s41598-018-32636-3 PMC6170442

[phy215833-bib-0014] Drozak, J. , Chrobok, L. , Poleszak, O. , Jagielski, A. K. , & Derlacz, R. (2013). Molecular identification of carnosine N‐methyltransferase as chicken histamine N‐methyltransferase‐like protein (hnmt‐like). PLoS One, 8, e64805. 23705015 10.1371/journal.pone.0064805 PMC3660329

[phy215833-bib-0015] Drozak, J. , Piecuch, M. , Poleszak, O. , Kozlowski, P. , Chrobok, L. , Baelde, H. J. , & De Heer, E. (2015). UPF0586 protein C9orf41 homolog is anserine‐producing methyltransferase. The Journal of Biological Chemistry, 290, 17190–17205. 26001783 10.1074/jbc.M115.640037 PMC4498059

[phy215833-bib-0016] Drozak, J. , Veiga‐Da‐Cunha, M. , Vertommen, D. , Stroobant, V. , & Van Schaftingen, E. (2010). Molecular identification of carnosine synthase as ATP‐grasp domain‐containing protein 1 (ATPGD1). The Journal of Biological Chemistry, 285, 9346–9356. 20097752 10.1074/jbc.M109.095505 PMC2843183

[phy215833-bib-0017] Dunnett, M. , & Harris, R. C. (1997). High‐performance liquid chromatographic determination of imidazole dipeptides, histidine, 1‐methylhistidine and 3‐methylhistidine in equine and camel muscle and individual muscle fibres. Journal of Chromatography. B, Biomedical Sciences and Applications, 688, 47–55. 9029312 10.1016/s0378-4347(97)88054-1

[phy215833-bib-0018] Dutka, T. L. , Lamboley, C. R. , Mckenna, M. J. , Murphy, R. M. , & Lamb, G. D. (2012). Effects of carnosine on contractile apparatus Ca(2)(+) sensitivity and sarcoplasmic reticulum Ca(2)(+) release in human skeletal muscle fibers. Journal of Applied Physiology, 1985(112), 728–736. 10.1152/japplphysiol.01331.2011 22174397

[phy215833-bib-0019] Everaert, I. , Mooyaart, A. , Baguet, A. , Zutinic, A. , Baelde, H. , Achten, E. , Taes, Y. , De Heer, E. , & Derave, W. (2011). Vegetarianism, female gender and increasing age, but not CNDP1 genotype, are associated with reduced muscle carnosine levels in humans. Amino Acids, 40, 1221–1229. 20865290 10.1007/s00726-010-0749-2

[phy215833-bib-0020] Everaert, I. , Stegen, S. , Vanheel, B. , Taes, Y. , & Derave, W. (2013). Effect of beta‐alanine and carnosine supplementation on muscle contractility in mice. Medicine and Science in Sports and Exercise, 45, 43–51. 22895378 10.1249/MSS.0b013e31826cdb68

[phy215833-bib-0021] Flancbaum, L. , Fitzpatrick, J. C. , Brotman, D. N. , Marcoux, A. M. , Kasziba, E. , & Fisher, H. (1990). The presence and significance of carnosine in histamine‐containing tissues of several mammalian species. Agents and Actions, 31, 190–196. 2085137 10.1007/BF01997607

[phy215833-bib-0022] Goncalves, L. S. , Kratz, C. , Santos, L. , Carvalho, V. H. , Sales, L. P. , Nemezio, K. , Longobardi, I. , Riani, L. A. , Lima, M. M. O. , Saito, T. , Fernandes, A. L. , Rodrigues, J. , James, R. M. , Sale, C. , Gualano, B. , Geloneze, B. , de Medeiros, M. H. G. , & Artioli, G. G. (2020). Insulin does not stimulate beta‐alanine transport into human skeletal muscle. American Journal of Physiology. Cell Physiology, 318, C777–C786. 32101455 10.1152/ajpcell.00550.2019

[phy215833-bib-0023] Goncalves, L. S. , Sales, L. P. , Saito, T. R. , Campos, J. C. , Fernandes, A. L. , Natali, J. , Jensen, L. , Arnold, A. , Ramalho, L. , Bechara, L. R. G. , Esteca, M. V. , Correa, I. , Sant'anna, D. , Ceroni, A. , Michelini, L. C. , Gualano, B. , Teodoro, W. , Carvalho, V. H. , Vargas, B. S. , … Artioli, G. G. (2021). Histidine dipeptides are key regulators of excitation‐contraction coupling in cardiac muscle: Evidence from a novel CARNS1 knockout rat model. Redox Biology, 44, 102016. 34038814 10.1016/j.redox.2021.102016 PMC8144739

[phy215833-bib-0024] Harris, R. C. , Tallon, M. J. , Dunnett, M. , Boobis, L. , Coakley, J. , Kim, H. J. , Fallowfield, J. L. , Hill, C. A. , Sale, C. , & Wise, J. A. (2006). The absorption of orally supplied beta‐alanine and its effect on muscle carnosine synthesis in human vastus lateralis. Amino Acids, 30, 279–289. 16554972 10.1007/s00726-006-0299-9

[phy215833-bib-0025] Harris, R. C. , Wise, J. A. , Price, K. A. , Kim, H. J. , Kim, C. K. , & Sale, C. (2012). Determinants of muscle carnosine content. Amino Acids, 43, 5–12. 22327512 10.1007/s00726-012-1233-y PMC3374101

[phy215833-bib-0026] Hoetker, D. , Chung, W. , Zhang, D. , Zhao, J. , Schmidtke, V. K. , Riggs, D. W. , Derave, W. , Bhatnagar, A. , Bishop, D. J. , & Baba, S. P. (2018). Exercise alters and beta‐alanine combined with exercise augments histidyl dipeptide levels and scavenges lipid peroxidation products in human skeletal muscle. Journal of Applied Physiology, 1985, 1767–1778. 10.1152/japplphysiol.00007.2018 PMC10392632 30335580

[phy215833-bib-0027] Kohen, R. , Yamamoto, Y. , Cundy, K. C. , & Ames, B. N. (1988). Antioxidant activity of carnosine, homocarnosine, and anserine present in muscle and brain. Proceedings of the National Academy of Sciences of the United States of America, 85, 3175–3179. 3362866 10.1073/pnas.85.9.3175 PMC280166

[phy215833-bib-0028] Lievens, E. , Van Vossel, K. , Van de Casteele, F. , Baguet, A. , & Derave, W. (2021). Sex‐specific maturation of muscle metabolites carnosine, creatine, and carnitine over puberty: A longitudinal follow‐up study. Journal of Applied Physiology, 1985(131), 1241–1250. 10.1152/japplphysiol.00380.2021 34473575

[phy215833-bib-0029] Mannion, A. F. , Jakeman, P. M. , Dunnett, M. , Harris, R. C. , & Willan, P. L. (1992). Carnosine and anserine concentrations in the quadriceps femoris muscle of healthy humans. European Journal of Applied Physiology and Occupational Physiology, 64, 47–50. 1735411 10.1007/BF00376439

[phy215833-bib-0030] Mcmanus, I. R. (1962). Enzymatic synthesis of anserine in skeletal muscle by N‐methylation of carnosine. The Journal of Biological Chemistry, 237, 1207–1211.

[phy215833-bib-0031] Painelli, V. S. , Nemezio, K. M. , Pinto, A. J. , Franchi, M. , Andrade, I. , Riani, L. A. , Saunders, B. , Sale, C. , Harris, R. C. , Gualano, B. , & Artioli, G. G. (2018). High‐intensity interval training augments muscle carnosine in the absence of dietary Beta‐alanine intake. Medicine and Science in Sports and Exercise, 50, 2242–2252. 30334920 10.1249/MSS.0000000000001697

[phy215833-bib-0032] Peters, V. , Klessens, C. Q. , Baelde, H. J. , Singler, B. , Veraar, K. A. , Zutinic, A. , Drozak, J. , Zschocke, J. , Schmitt, C. P. , & de Heer, E. (2015). Intrinsic carnosine metabolism in the human kidney. Amino Acids, 47, 2541–2550. 26206726 10.1007/s00726-015-2045-7 PMC4633449

[phy215833-bib-0033] Rezende, N. S. , Swinton, P. , de Oliveira, L. F. , da Silva, R. P. , da Eira Silva, V. , Nemezio, K. , Yamaguchi, G. , Artioli, G. G. , Gualano, B. , Saunders, B. , & Dolan, E. (2020). The muscle carnosine response to Beta‐alanine supplementation: A systematic review with Bayesian individual and aggregate data E‐max model and meta‐analysis. Frontiers in Physiology, 11, 913. 32922303 10.3389/fphys.2020.00913 PMC7456894

[phy215833-bib-0034] Saunders, B. , Painelli, V. D. E. S. , Oliveira, L. F. D. E. , Silva, V. D. A. E. , Silva, R. P. D. A. , Riani, L. , Franchi, M. , Goncalves, L. S. , Harris, R. C. , Roschel, H. , Artioli, G. G. , Sale, C. , & Gualano, B. (2017). Twenty‐four weeks of beta‐alanine supplementation on carnosine content, related genes, and exercise. Medicine and Science in Sports and Exercise, 49, 896–906. 28157726 10.1249/MSS.0000000000001173

[phy215833-bib-0035] Smith, E. C. (1938). The buffering of muscle in rigor; protein, phosphate and carnosine. The Journal of Physiology, 92, 336–343. 16994977 10.1113/jphysiol.1938.sp003605 PMC1395289

[phy215833-bib-0036] Yamaguchi, G. C. , Nemezio, K. , Schulz, M. L. , Natali, J. , Cesar, J. E. , Riani, L. A. , Goncalves, L. S. , Moller, G. B. , Sale, C. , Mhg, D. E. M. , Gualano, B. , & Artioli, G. G. (2021). Kinetics of muscle carnosine decay after beta‐alanine supplementation: A 16‐wk washout study. Medicine and Science in Sports and Exercise, 53, 1079–1088. 33148972 10.1249/MSS.0000000000002559 PMC8048732

[phy215833-bib-0037] Zhao, J. , Conklin, D. J. , Guo, Y. , Zhang, X. , Obal, D. , Guo, L. , Jagatheesan, G. , Katragadda, K. , He, L. , Yin, X. , Prodhan, M. A. I. , Shah, J. , Hoetker, D. , Kumar, A. , Kumar, V. , Wempe, M. F. , Bhatnagar, A. , & Baba, S. P. (2020). Cardiospecific overexpression of ATPGD1 (carnosine synthase) increases histidine dipeptide levels and prevents myocardial ischemia reperfusion injury. Journal of the American Heart Association, 9, e015222. 32515247 10.1161/JAHA.119.015222 PMC7429021

